# Primordial Germ Cells: Current Knowledge and Perspectives

**DOI:** 10.1155/2016/1741072

**Published:** 2015-11-09

**Authors:** Aleksandar Nikolic, Vladislav Volarevic, Lyle Armstrong, Majlinda Lako, Miodrag Stojkovic

**Affiliations:** ^1^Center for Molecular Medicine and Stem Cell Research, Faculty of Medical Sciences, University of Kragujevac, 69 Svetozara Markovica Street, 34000 Kragujevac, Serbia; ^2^Institute of Genetic Medicine, Newcastle University, The International Centre for Life, Central Parkway, Newcastle upon Tyne NE1 3BZ, UK; ^3^Spebo Medical, Norvezanska 16, 16 000 Leskovac, Serbia

## Abstract

Infertility is a condition that occurs very frequently and understanding what defines normal fertility is crucial to helping patients. Causes of infertility are numerous and the treatment often does not lead to desired pregnancy especially when there is a lack of functional gametes. In humans, the primordial germ cell (PGC) is the primary undifferentiated stem cell type that will differentiate towards gametes: spermatozoa or oocytes. With the development of stem cell biology and differentiation protocols, PGC can be obtained from pluripotent stem cells providing a new therapeutic possibility to treat infertile couples. Recent studies demonstrated that viable mouse pups could be obtained from *in vitro* differentiated stem cells suggesting that translation of these results to human is closer. Therefore, the aim of this review is to summarize current knowledge about PGC indicating the perspective of their use in both research and medical application for the treatment of infertility.

## 1. Introduction

Today, in the second decade of this millennium, infertility remains a global condition with a high prevalence of occurrence [1, 2]. Boivin et al. [3] revealed that the rate of 12-month prevalence ranges from 3.5% to 16.7% in more developed and from 6.9% to 9.3% in less-developed countries, with an average prevalence of 9%. The diagnosis of infertility may become a cause of life crisis in many couples, so it is necessary to develop mechanisms to overcome temporary or permanent loss of fertility and the possibility to have a biological child [1]. For infertile couples that are unable to have a child by other treatments, artificial reproductive technique (ART) and the possibility to derive gametes from stem cells offer potential reproductive strategies to individuals who are infertile due to injuries, exposure to toxicants, or immune-suppressive treatments and suffer from gonadal insufficiency due to premature ovarian failure or azoospermia, reproductive aging, and idiopathic cases of poor gamete quality [4, 5].

In most multicellular organisms, germ cells are the origin of new organisms that provide the inheritance of the genetic and epigenetic information in the following generations [6]. The germ cell lineage is the source of totipotency, providing the creation of new organisms [7]. In the 19th century, August Weismann published the hypothesis according to which the presence of preformed germ cell determinants (germplasm) are inherited only through the germ cells ensuring the totipotency and continuity of the germ line [8]. This has been demonstrated in invertebrates and lower vertebrates, where the germplasm is required for germ cell formation, while in mammals, this process involves epigenetic mechanisms and not preformation. That regulated event, in which some environmental influences play a crucial role, and totipotency is maintained through the germ line, allowing further development in the following generation.

Germ cells undergo two significantly different developmental phases [9]. The first phase occurs during early embryogenesis, when primordial germ cells (PGCs) are formed and actively migrate to the gonadal ridge [10, 11]. In the second phase, the germ cells receive appropriate signals from their environment and initiate one of two distinct programs of controlled cell division, meiosis, and differentiation-oogenesis or spermatogenesis, to form gametes. The molecular basis of both processes and early germ cell development is very well understood in two species,* Drosophila* and* Caenorhabditis elegans*, where systematic genetic reviews have identified many of the genes required in this process [12–15]. PGCs in humans have not been intensely investigated because of the technical and ethical obstacles to obtaining such cells from early embryos [16]. The greater part of our knowledge of mammalian PGC specification has been obtained from studies using early mouse embryos, in which the germ cell fate is induced in pluripotent proximal epiblast cells soon after implantation in the uterine wall [17].

In this review we summarize current knowledge about mammalian PGC and indicate the perspective of their use in research and to generate mature gametes that could be used in treatment of human infertility.

## 2. Origin and Development of Primordial Germ Cells

In mammals, the origin of the germ cell lineage in embryogenesis was initially unclear due to the absence of the characteristic germplasm present in the egg as seen in other organisms such as* X. laevis* and* D. melanogaster* [17]. PGCs were first identified in mammals by Chiquoine in 1954 [18]. He found a population of germ cell lineage cells capable of generating both, oocytes and spermatozoa at the base of the emerging allantois at E7.25 in the endoderm of the yolk sac of mouse embryos, immediately below the primitive streak, identified by high alkaline phosphatase (AP) activity. While ethical constraints limit our knowledge of the specification of human PGC, it is clear that common signaling pathways operate across mammals and possibly all vertebrates [19]. Based upon staining of AP different studies have suggested diverse sites of origin for the PGC including the posterior primitive streak (reviewed in [20]). The founder population of germ cells is few in number and deeply which causes major difficulties in studying the genetic basis for the specification of the germ cell lineage [8]. Germ cells, soon after their lineage restriction, acquire morphology which would reflect underlying unique molecular features. Saitou et al. have established a system to identify key factors that determine germ cell fate in mouse and to understand the distinctive features that the germ cells acquire at the molecular levels [21]. They dissected out an embryonic region with around 300 cells that contained the founder germ cells at E7.5 (EB stage) and dissociated it into single cells. These cells are morphologically similar but fall into two classes distinguishable by differential expression of two germ cell-specific genes,* Stella* and* Fragilis* [8]. The cluster of 300 cells demonstrates universal expression of* Fragilis* but* Stella* expression is restricted to a subset of cells within the centre of the cluster. Therefore, both genes appear to have major roles in germ cell development and their ability to differentiate [21].* Stella* is the first gene to be expressed in the population of cells considered to be lineage restricted germ cells [8] that also show high expression of tissue nonspecific AP (*Tnap*), a gene for AP activity of PGC [22].

Migrating germ cells continue to show strong and specific expression of* Stella* but exhibit strong repression of all the homeobox genes examined (*Hoxb1*,* Hoxa1*,* Evx1*, and* Lim1*), despite high levels of expression of these genes in the neighbouring somatic cells [8]. Since the role of homeobox genes is to specify the regional identity of cells along the body axis or induce differentiation of cells towards specific somatic cell lineages, this suggests that the founder germ cells acquire the ability to avoid somatic specification by preventing or suppressing homeobox gene expression. This could be one of the key features that mammalian germ cells possess that allows them to maintain or regain totipotency and differ from other surrounding cells in the niche. This concept is supported by continued expression of* Oct4* and other pluripotency genes in germ cells [23].

In mouse, the specification of germline begins around E6.25 in the proximal epiblast in a small population of cells identified by expression of* Blimp1* (B-lymphocyte-induced maturation protein 1)/*Prdm1* (Pr domain containing protein 1) [24] and* Prdm14* [25]. Interestingly, Blimp1 and Prdm14 have distinct binding patterns relative to promoters [26] and Blimp1 has a dominant role for PGC specification. Blimp1 is important for the repression of almost all the genes normally downregulated in PGC with respect to their somatic neighbours, as well as for the restoration of pluripotency and epigenetic reprogramming. Conversely, Prdm14 regulates the restoration of pluripotency and epigenetic reprogramming independently from Blimp1 and defines a novel genetic pathway with strict specificity to the germ cell lineage [27]. Expression of these two factors starts independently in a small number of cells of the proximal posterior epiblast at the beginning of the early-streak stage [28]. These cells increase in number and form PGC with AP activity and* Stella* (also known as* Dppa3* or* Pgc7*) expression [21, 29]. PGCs in the mouse are induced during gastrulation by bone morphogenetic protein (BMP) signaling and as yet unidentified signal(s) from the extraembryonic ectoderm and visceral endoderm to underlying pluripotent epiblast cells at E6.5 [30]. This induction results in* Blimp1*/*Prdm1* mediated transcriptional regulation of epiblast cells which promotes the expression of PGC-specific genes, such as* Stella*, and represses the expression of somatic cell genes such as members of the* Hox* gene family [24, 31]. It is still unknown whether the Smads activate* Blimp1* and* Prdm14* transcription directly or indirectly [7]. Determination of the element(s) responsible for* Blimp1* expression in the epiblast in response to BMP4 and examination of whether or not the Smads directly bind to and control Blimp1 will be crucial to provide a definitive answer to this question. Induction of PGC seems to require BMP4 or BMP8b alone or in combination indicating that signalling for various BMPs occurs through separate receptors. In mice, a number of other factors which have been implicated in specification and maintenance of PGC occur at around E6.25, marked by the sequential expression of two transcription factors* Blimp1*/*Prdm1* and* Prdm14* in response to BMPs [32].* Blimp1*,* Prdm14*, and* Stella*-positive PGC integrate key events to repress a somatic mesodermal differentiation program in PGC [7]. WNT signalling is also essential for the PGC fate, possible through posttranscriptional interaction with suggesting that the WNT signaling may act posttranscriptionally on BMP signalling components [7]. WNT signaling can stabilize Smad1 by inhibiting GSK3-mediated phosphorylation of its linker region thereby preventing its degradation [33] but the tyrosine-kinase receptor c-kit and its ligand, stem cell factor, are essential for the maintenance of PGC in both sexes [19].

## 3. Migration of Primordial Germ Cells

In contrast to* D. melanogaster* and zebrafish, little is known about PGC migration and initiation of that process in mice [33]. Soon after specification, the cells begin to exhibit polarized morphology and cytoplasmic extensions and initiate migration through the primitive streak into the adjacent posterior embryonic endoderm, extraembryonic endoderm, and allantois [20]. The first step in mouse PGC migration is the movement of cells from the posterior primitive streak to the endoderm at E7.5. Between E8.5 and E13.5, the* Tnap* positive PGCs proliferate and migrate via the hindgut endoderm and mesentery followed by bilateral migration to the genital ridges, after which they can enter into meiosis in the female or mitotic arrest in the male and initiate differentiation into either oocytes or spermatozoa [16, 34]. During migration, the PGC population doubling time is fairly uniform at about 16 hours between 8.5 and 13.5 days. E13.5 mouse embryos should have about 24000 PGCs in their genital ridges [35]. In this migratory phase, PGCs undergo extensive genome reprogramming and alteration of epigenetic information, such as DNA methylation and histone modification patterns, and the erasure of gene imprinting that may be essential for restoring totipotency to the germ cell lineage [16].

**Figure 1:**
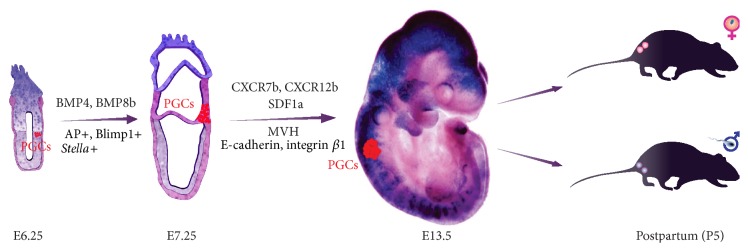
Time schedule and signals involved in the regulation of primordial germ cells (PGCs) migration and colonization in mice.

Currently, there is no evidence for sex-specific differences during PGC migration in any organism. A subset of germ cells in the gonad acquires the ability to function as germline stem cells, which undergo meiosis to produce sperm and eggs and promote the next generation of embryonic development and PGC migration. Vasa protein is an essential component of germplasm and represents a poorly understood complex of RNA and proteins that is required for germ cell determination. Null mutation leads to sterility in female mice resulting from severe defects in oogenesis [10]. In humans,* VASA* expression begins at the end of the migratory phase of PGC development [9]. Tilgner et al. generated and characterized human embryonic stem cell (hESC) lines with a construct in which expression of the pEGFP-1 gene was driven by a DNA sequence representing the* VASA* reporter [36]. They demonstrated that hESC could be used as a development model system for PGC specification under* in vitro* conditions. Also, they were able to establish a small number of the presumed female PGCs isolated on the basis of* VASA* promoter driven GFP fluorescence. These results and the fact that specific expression of* Vasa* in the germ cell lineage during colonization of the gonadal ridge suggest that Vasa is required to maintain the functionality of germ cells. For instance, male mice homozygous for a targeted mutation of the mouse* Vasa* ortholog* Mvh* are sterile and exhibit severe defects in spermatogenesis while homozygous females are fertile [37, 38]. Other signals (Figure 1) involved in the regulation of PGC migration and colonization are the adhesion molecule* E-cadherin* [39] and extracellular matrix molecule integrin *β*1 [40, 41]. Unfortunately, the precise function of these factors and signalling pathways remain to be explained.

Migratory PGCs maintain a genomic program associated with pluripotency. They express core pluripotency genes (*Oct4*,* Nanog*, and* Sox2*) and are able to form teratomas after injection into postnatal mouse testes [6, 42, 43]. Besides these, migratory PGCs express stage-specific embryonic antigen 1 (SSEA1) [16]. Upon arrival in the gonad, germ cell-specific RNA binding protein DAZL (deleted in azoospermia-like) is essential for developing PGC [44]. Many studies revealed that the DAZL functions as a translational enhancer [45–47]. Knockout for target mRNA binding partners of DAZL (Mvh, Scp3, and Tex19.1) resulted in severe phenotypic changes [48–50] suggesting that DAZL may have additional roles during the PGC stage of mammalian gametogenesis. DAZL is also a gatekeeper of apoptosis in PGC and regulates the expression of key Caspases acting as an elegant fail-safe mechanism that prevents stray PGC from forming teratomas and eliminating aberrant PGC [51]. In the absence of DAZL, the germ cells fail to develop beyond the PGC stage as shown by continued expression of pluripotency markers. These findings suggest that DAZL is a “licensing factor” required for sexual differentiation of PGC [52]. Genes that participate in PGC development are summarized in Table 1.

Postmigration PGCs, marked by the expression of several RNA binding proteins such as MVH, DAZL, and NANOS3, undergo sexual dimorphic development [46–53]. In mice, female germ cells quickly initiate meiosis and arrest at meiosis I stage, while male ones mitotically divide for several rounds and then enter a quiescent stage when they are known as gonocytes. Specifically, the PGCs upregulate a set of genes that enable them to undergo sexual differentiation and gametogenesis while suppressing their pluripotency program [54]. In the female XX embryo, the PGCs continue to proliferate and subsequently enter into the prophase I of meiotic divisions [35]. Hereafter, they are arrested at the diplotene stage of prophase I of meiosis. After birth, the gonocytes are surrounded by cells from the cortical interstitial layer and become primary oocytes in primordial follicles, thereby ending precursor proliferative potential and arresting their development process until puberty. After puberty, hormonal stimulation during ovulation causes the maturation and release of oocytes from the ovary into the oviduct followed by completion of the first meiotic division with concomitant extrusion of the first polar body. Upon fertilization with a haploid spermatozoon, the oocyte completes the second meiotic division and extrudes the second polar body [6, 55, 56].

In contrast to those in the female, XY PGCs enter into mitotic arrest upon entry into the genital ridges and stay quiescent in the *G*_0_/*G*_1_ phase of the cell cycle for the remaining embryonic period as a prospermatogonium, while retaining a proliferative precursor potential [57]. Around day 5 postpartum, many of prospermatogonia resume active proliferation, while some migrate to the basement membrane of the seminiferous tubule and form tight junctions with Sertoli cells thereby forming spermatogonial stem cells (SSC) incorporated into their appropriate niche. Thus, compared with the very limited size of the oocyte pools, the spermatozoa can be obtained from differentiation of SSC. In culture, germline stem cells bearing the abilities for long-term proliferation and for spermatogenesis upon transplantation into testes are established in the presence of GDNF (glial cell-derived neurotrophic factor), most readily from neonatal testes [6, 55, 56].

## 4. Derivation of PGC from Pluripotent Cells

PGC can be isolated from whole mouse embryos at E6.0 in serum- and feeder-free conditions in suspension [7]. Under such conditions leukaemia inhibitory factor has an activity to suppress the induction of goosecoid, a marker for the mesendoderm, which suggests that* Blimp1* expression in the serum-free medium might reflect a differentiation of epiblast cells into a mesendodermal lineage. The data indicate that essentially all epiblast cells at E6.0, if separated from visceral endoderm a source for inhibitory signals, are able to express* Blimp1* in response to BMP4 alone (Figure 1). Until now, evidence has been provided that isolated epiblasts can be induced to form* Blimp1*,* Prdm14*, and AP-positive PGC-like cells after 36 hours in a serum-free culture with BMP4 [58].

**Figure 2:**
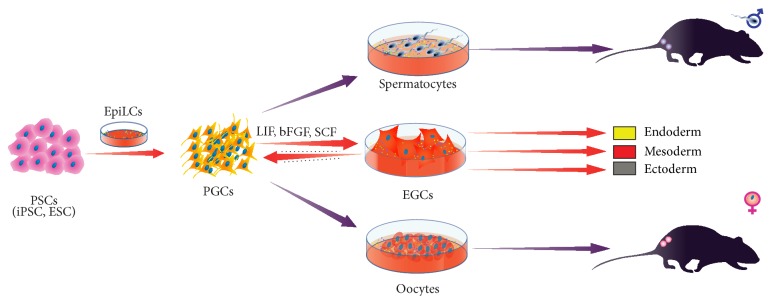
Making gametes from pluripotent stem cells (PSC). Primordial germ cells (PGCs), embryonic germ cells (EGCs).

Meanwhile differentiation of hESC to PGC has substantial potential as a method to examine the mechanisms of normal and abnormal development of human germline. To derive larger numbers of human PGCs from hESC Tilgner et al. [16] have developed an* in vitro* growth system that demonstrates the usefulness of SSEA1 to enrich a population of putative PGC. The SSEA1-positive cells share many characteristics with* ex vivo* PGC, such as the expression of key genes (*VASA*,* OCT4*, and* STELLA*), but their cell-cycle status differs from previous observations in embryonic mice, indicating that PGCs from E9.0 were largely arrested in G2/M, while the SSEA1-positive population is largely in S-phase, which suggests that the majority of the cells may still be in their mitotic expansion phase. Therefore, targeted differentiation of human pluripotent stem cells (PSC) offers an excellent opportunity to investigate the mechanisms involved in maturation of PGC. However, the limited number of publications indicates that the derivation of germ cells from PSC is still an immature technology [59–61]. The first reported study of induction of mouse germ cells from PSC was study by Hübner et al. [60] but Hayashi et al. [62] were the first to describe the high efficiency two-step procedure to obtain PGC-like cells (PGCLC) from mouse ESC and iPSC. In this procedure, ESC and iPSC were first induced into epiblast-like cells that were subsequently induced to PGCLC [62] and later ESC could be differentiated towards germ cells [63]. Chuma et al. [42] transplanted PGC in testis and obtained mature sperm whereas Matoba and Ogura [64] reported that PGCs isolated from E12.5 male foetus under the kidney capsule yield spermatids (Figure 2). The milestone of these studies is the birth of healthy offspring. Similar to Matoba and Ogura Hashimoto et al. [65] reported that PGCs isolated from female foetus when transplanted under the ovarian bursa or kidney capsule result in functional oocytes. Therefore, we are a step closer to obtaining human gametes from* in vitro* produced PGC using ESC or iPSC [62, 66, 67] but in human some obstacles remain: (i) how to make the PGC to convert to mature oocyte without transplantation and (ii) how to repeat the mouse work in humans to produce PGC for infertility treatment. Nevertheless, the advantage of using hESC and patient-specific iPSC is the possibility to decipher the mechanisms involved in differentiation and maturation of human gametes with the aim of completely translating gene expression profiling of these cells. This can be confirmed by previous publication [68] with clear evidence that the* SOX17* is the key regulator of human PGC fate. The study revealed that the* Blimp1* is downstream of* SOX17* and represses endodermal and other somatic genes during specification of human PGC, which was unexpected since* Sox17* does not have role in specification of mouse PGC. Once we have in our hands the protocol to drive differentiation of human iPSC towards functional gametes we will be able to produce patient-specific oocytes and spermatozoa under controlled* in vitro* conditions.

## 5. Conclusion

The possibility of generating mature gametes from PGC represents an area of investigation that provides more insight into signalling pathways of gametogenesis and reproductive dysfunction in humans. Very recent progress in work with targeted differentiation of iPSC offers tailor-made/personalized iPSC therapies also for the treatments of azoospermia in males or primary ovarian insufficiency in females [69–73]. However, problems in derivation of sperm cells and oocytes under* in vitro* conditions in human remain: the differentiation of germ cells is dependent on the somatic environment rather than the sex chromosome content of the germ cell. Therefore, the demonstration of gametogenesis from cultures of differentiating mouse ESC and iPSC suggests that “artificial” human gametes may also be obtained in this way, and the live birth of mouse pups suggests that the human cells may also be functional, although the successful demonstration of human gamete formation in this way is still awaited, but already followed by numerous ethical discussions. Nevertheless, obtaining of human viable spermatozoa and oocytes under* in vitro* conditions would for sure open in reproductive medicine new horizons to a new form of infertility treatment.

## Figures and Tables

**Table 1:** Summarized genes that participate into PGC development.

Full name	Alternate names	Time of expression	Location of coded protein	References
Tissue nonspecific AP (Tnap)		E7.25-14	At the cell surface, linked to the cell membrane via a phosphatidylinositol glycan linkage	[22]

*Stella *	*Developmental pluripotency-associated 3 or Dppa3 *or *Pgc7 *	E7.5	Protein that may shuttle between the nucleus and cytoplasm	[8, 21]

*Fragilis *		E7.5	Transmembrane protein	[8, 21]

B-lymphocyte-induced maturation protein 1 (*Blimp1*)	Pr domain containing protein 1 (*Prdm1*)	E6.25	Cytoplasmic transcriptional repressor	[24]

*Prdm14 *	Pr domain containing protein 14	E6.25	Transcriptional regulator	[25]

Mouse *Vasa* ortholog (*Mvh*)		E13.5(10.5) end of the migratory phase of PGC development	Cytoplasm	[9]

Deleted in azoospermia-like (DAZL)		E12.5(11.5)	Cytoplasmic protein	[44–52]
